# Surgical management of Miescher’s cheilitis: a case report

**DOI:** 10.3205/iprs000159

**Published:** 2021-07-22

**Authors:** Juan Cámara-Pérez, José Carlo Zapata-Negreiros, Pedro Enrique Alonso, Fernando Leiva-Cepas

**Affiliations:** 1 Department of Plastic and Reconstructive Surgery, Hospital Universitario Reina Sofia, Cordoba, Spain; 2 Department of Pathology, Hospital Universitario Reina Sofía, Córdoba, Spain; 3 Department of Morphological Sciences, Medicine & Nurse School, University of Cordoba, Cordoba, Spain; 4 Research Group on Muscle Regeneration, University of Cordoba, Cordoba, Spain

**Keywords:** Miescher’s cheilitis, Melkersson-Rosenthal syndrome, cheiloplasty

## Abstract

Miescher’s cheilitis, also known as cheilitis granulomatosa, is an infrequent disease characterized by chronic recurrent swelling of one lip or both lips. It is considered as one of the three main symptoms of the triad of the Melkersson-Rosenthal syndrome, although in many cases it may develop monosymptomatically.

The initial management is based on the administration of corticoids, followed in many cases by the use of other systemic treatments. Nevertheless, because recurrence is quite frequent, surgery remains in many cases as the only definitive treatment.

In this report we present the case of a Caucasian woman with Miescher’s cheilitis who was successfully surgically managed.

## Zusammenfassung

Cheilitis granulomatosa (Miescher) ist eine seltene Erkrankung, die sich durch chronisch wiederkehrende Schwellungen einer oder beiden Lippen auszeichnet. Sie wird als eine von drei Symptomen der Triade des Melkersson-Rosenthal-Syndroms betrachtet. Trotzdem kann sie sich monosymptomatisch entwickeln.

Die anfängliche Behandlung beruht auf Kortikoid-Therapie, gefolgt von anderen systemischen Medikamenten. Da trotzdem häufig ein Wiederauftreten vorkommt, bleibt eine Operation in vielen Fällen die einzige wirksame Behandlung.

In diesem Fallbericht wird die erfolgreiche chirurgische Behandlung einer Patientin mit Cheilitis granulomatosa vorgestellt.

## Introduction

Miescher’s cheilitis is a rare disease characterized by chronic recurrent swelling of one lip or both lips. It was first described by Alfred Guido Miescher in 1945 [1]. It is considered as one of three main symptoms of the Melkersson-Rosenthal syndrome, although it might present monosymptomatically. Histologically it is described as noncaseating granulomatous disease [2].

The etiology remains unclear. However, it has been associated to other factors such as allergic disorders, immunity alterations, microbacterial factors or UVB photosensitivity [3]. In addition, it has also been related to Crohn’s disease [4]. 

Corticoids are considered as the first-step treatment, although other drugs such as clofazimine or minociclyne might be useful is some cases [5]. However, because of its tendency toward recurrence, surgical management remains as the main option in multiple cases.

## Case description

A 32-year-old Caucasian woman was referred to the Plastic and Reconstructive Surgery Department of Hospital Universitario Reina Sofía in Córdoba, Spain, due to inferior lip swelling since two years before.

Hypertension, morbid obesity, and obstructive sleep apnea syndrome were the only remarkable diseases found through her medical history and her family history was not relevant. Any allergic etiology was discarded.

**Figure 1:**
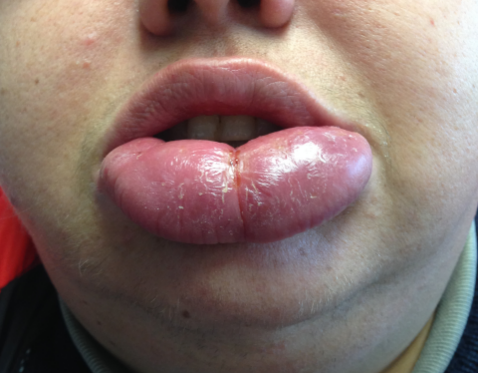
Preoperative photograph in which a great enlargement of the lower lip can be seen.

Physical examination showed local swelling limited to the lower lip, which was firmly elastic and painless (Figure 1 (Fig. 1)). No other symptoms of Melkersson-Rosenthal syndrome, such as fissured or plicated tongue or peripheral nerve paralysis, were present. Neither lymph node swelling, tonsils, nor fever were observed during physical exam. 

There was not any finding through blood examination such as leucocytosis, high levels of C-reactive protein or elevated erythrocyte sedimentation rate (ESR), which could suggest an infectious process.

CT-scan showed neither pulmonary nor digestive alterations. 

Because other medical treatments were ineffective and due to the increasing concern of the patient, surgical management was decided.

**Figure 2:**
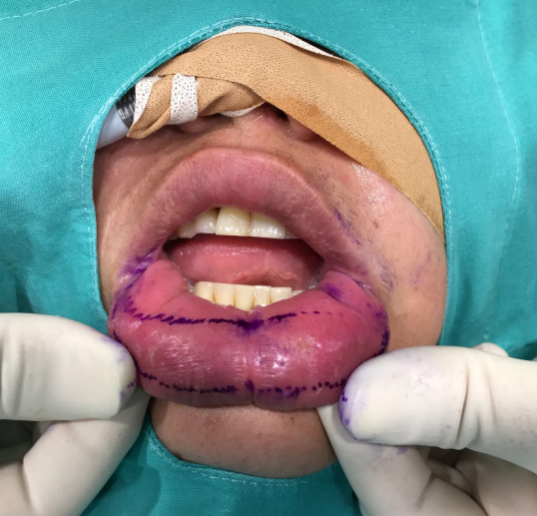
Intraoperative photograph showing the designed incisional pattern

**Figure 3:**
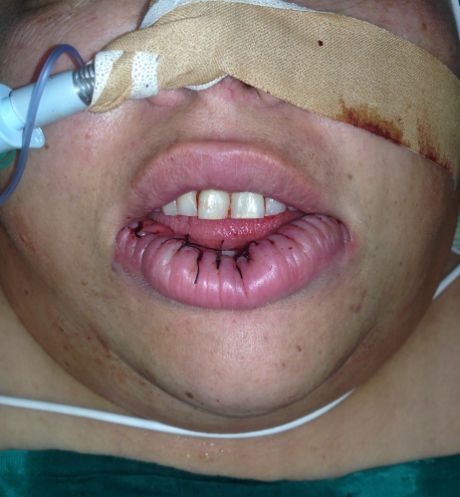
Immediate postoperative result

She was operated under general anesthesia. We undertook reducing cheiloplasty through partial bermellectomy of the lower lip, using a nearly semilunar incision (Figure 2 (Fig. 2)). Layered suture was then performed (Figure 3 (Fig. 3)). 

**Figure 4:**
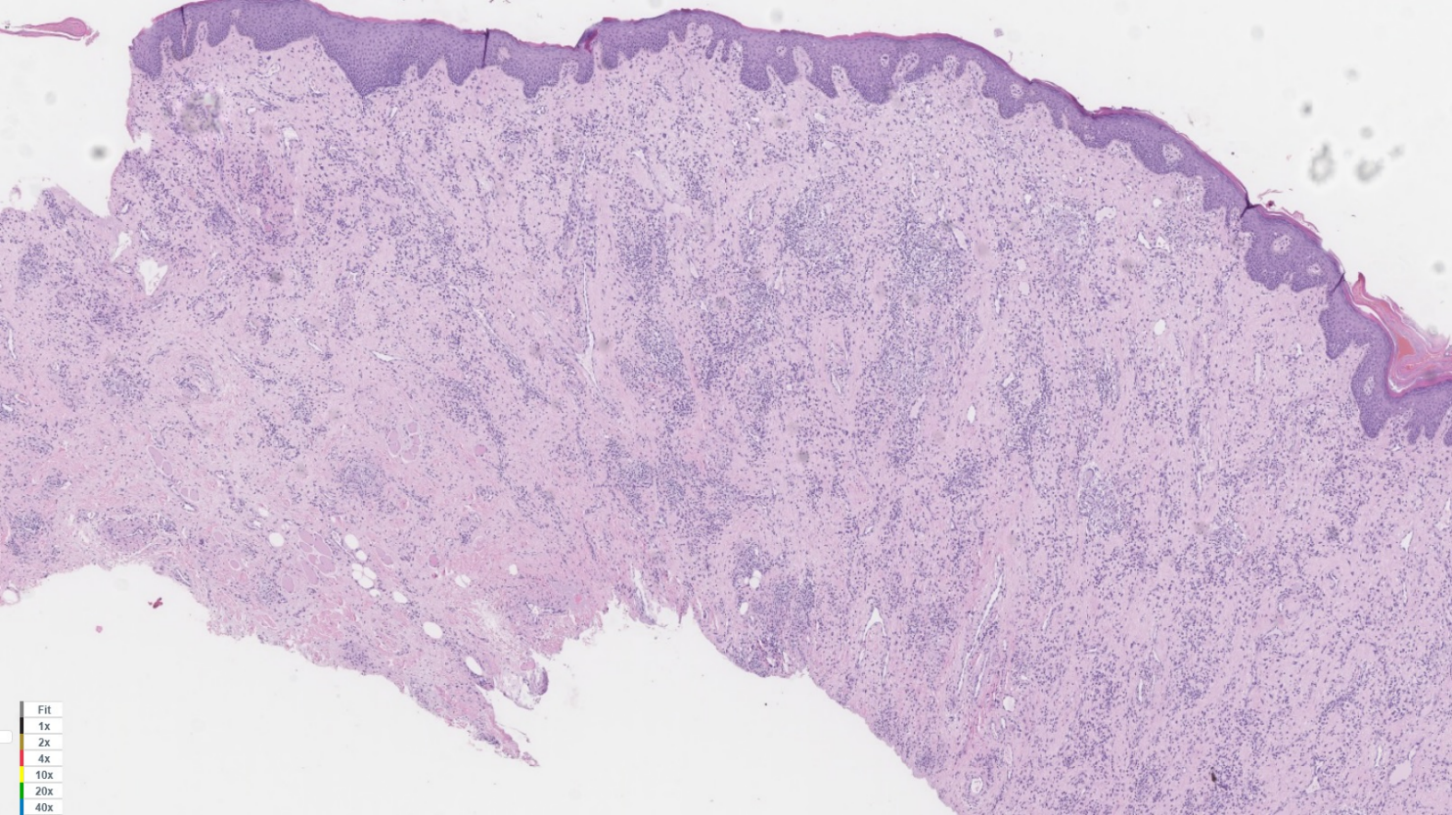
In this histological image, a mucous layer with chronic lymphoplasmacitic infiltrate can be seen, with presence of noncaseating granulomas. Hematoxylin and eosin (H&E). 2x.

**Figure 5:**
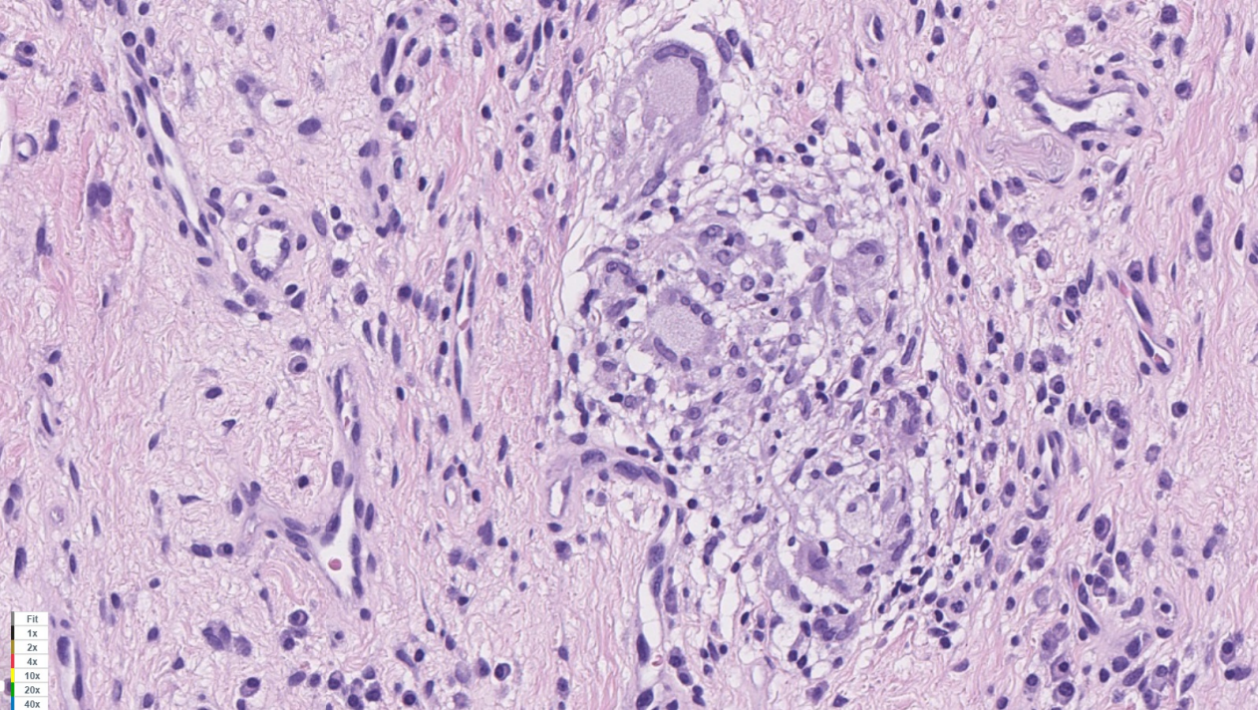
In this histological image, a mucous layer with chronic lymphoplasmacitic infiltrate can be seen, with presence of noncaseating granulomas. Hematoxylin and eosin (H&E). 20x.

A histopathological study of the surgical specimens was undertaken, showing superficial and deep chronic perivascular inflammation associated to granulomas, consistent with orofacial granulomatosis (cheilitis granulomatosa) (Figure 4 (Fig. 4), Figure 5 (Fig. 5)).

**Figure 6:**
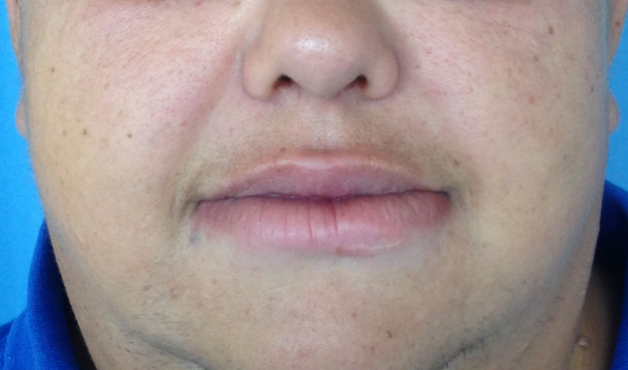
Final result after 1-year follow-up. Normal lower lip size has been achieved, in an adequate proportion to the upper one.

After the post-operative period, the patient reported high satisfaction with the result, both functional and aesthetical. During the follow-up period of 1 year no symptom of local recurrence was found (Figure 6 (Fig. 6)).

## Discussion

Miescher’s cheilitis, also known as granulomatous cheilitis, so named after Alfred Guido Miescher, who first described it in 1945 [1], is considered as one of the three main symptoms of the Melkersson-Rosenthal syndrome, in addition to lingula plicata and paralysis of the facial nerve [6]. However, it develops alone in up to 28% of the patients [7]. The clinical manifestations involve recurrent swelling of the lip, most frequently the upper one, being normally self-limited at the beginning, but with progressive remaining induration [8].

The etiology of the disease is still unknown, although many factors have been proposed, such as genetic predisposition, local alteration of innate immunity, allergic factors, microbial factors, hypersensitivity to ultraviolet B radiation [3].

Although MRI could be useful to differentiate the etiology of facial nerve palsy (Bell’s palsy vs. Melkersson-Rossenthal) [9], its utility is limited only to this symptom. In case of limb thickness, such in case of Miescher cheilitis, MRI is unable to give enough specific information to obtain a definitive diagnosis [10], [11], which is achieved only after biopsy and histological examination. 

The pathological study plays an important role in the diagnosis of the disease, showing presence of lymphedema and noncaseating granulomas in the lamina propria with chronic inflammatory response [2].

Granulomatous disorders such as sarcoidosis or tuberculosis should be included in the differential diagnosis, as well as Crohn’s disease. In fact, it has been proposed that Miescher’s Cheilitis could be included as one extraintestinal manifestation of the Crohn’s spectrum [12]. 

Corticoids, both intralesional or systemic, are considered as the first-line treatment. However, other drugs have also been proposed as an alternative, including clofazimine, hydroxicloroquine or sulfasalazine [13]. In other cases, as in our patient, surgery remains finally as the best option. In fact, medical treatment results are usually only partially successful, their effect is normally limited to the acute phase and the pathology often recurs [14]. However, surgical treatment has shown good long-term results [15]. Although it may be considered as more aggressive, it allows a reliable solution for the disease, with positive both functional and aesthetical outcome self-perceived by the patient. This is especially relevant in monosymptomatic cases of the Melkersson-Rosenthal syndrome, limited to lip swelling, as surgery allows a reliable and cosmetic solution for the morphological manifestation of the disease [16]. Because of that, as a conclusion, we must consider surgical management as a rapid and effective treatment in some cases of Miescher’s syndrome, particularly in recurrent cases or when cosmetic outcome is an important concern for the patient.

## Notes

### Competing interests

The authors declare that they have no competing interests.

### Informed consent 

Informed consent was previously obtained. Permission of the patient to publish the photographs was given.
